# Inhibition of mitochondrial complex I improves glucose metabolism independently of AMPK activation

**DOI:** 10.1111/jcmm.13432

**Published:** 2017-11-06

**Authors:** Wo‐Lin Hou, Jun Yin, Miriayi Alimujiang, Xue‐Ying Yu, Li‐Gen Ai, Yu‐qian Bao, Fang Liu, Wei‐Ping Jia

**Affiliations:** ^1^ Department of Endocrinology and Metabolism Shanghai Jiao Tong University Affiliated Sixth People's Hospital Shanghai China; ^2^ Shanghai Clinical Center for Diabetes Shanghai Clinical Center for Metabolic Diseases Shanghai China; ^3^ Shanghai Key Laboratory of Diabetes Mellitus Shanghai Diabetes Institute Shanghai China

**Keywords:** respiratory chain complex I, AMPK, glycolysis, gluconeogenesis

## Abstract

Accumulating evidences showed metformin and berberine, well‐known glucose‐lowering agents, were able to inhibit mitochondrial electron transport chain at complex I. In this study, we aimed to explore the antihyperglycaemic effect of complex I inhibition. Rotenone, amobarbital and gene silence of NDUFA13 were used to inhibit complex I. Intraperitoneal glucose tolerance test and insulin tolerance test were performed in db/db mice. Lactate release and glucose consumption were measured to investigate glucose metabolism in HepG2 hepatocytes and C2C12 myotubes. Glucose output was measured in primary hepatocytes. Compound C and adenoviruses expressing dominant negative AMP‐activated protein kinase (AMPK) α1/2 were exploited to inactivate AMPK pathway. Cellular NAD ^+^/NADH ratio was assayed to evaluate energy transforming and redox state. Rotenone ameliorated hyperglycaemia and insulin resistance in db/db mice. It induced glucose consumption and glycolysis and reduced hepatic glucose output. Rotenone also activated AMPK. Furthermore, it remained effective with AMPK inactivation. The enhanced glycolysis and repressed gluconeogenesis correlated with a reduction in cellular NAD ^+^/NADH ratio, which resulted from complex I suppression. Amobarbital, another representative complex I inhibitor, stimulated glucose consumption and decreased hepatic glucose output *in vitro*, too. Similar changes were observed while expression of NDUFA13, a subunit of complex I, was knocked down with gene silencing. These findings reveal mitochondrial complex I emerges as a key drug target for diabetes treatment. Inhibition of complex I improves glucose homoeostasis *via* non‐AMPK pathway, which may relate to the suppression of the cellular NAD ^+^/NADH ratio.

## Introduction

Mitochondrium, an important cell organelle, plays a dominant role in energy metabolism. Electron transport chain, also called respiratory chain, is located in the inner membrane of mitochondria and closely related with the production of adenosine triphosphate (ATP). The respiratory chain consists of four linked membrane protein complexes named complex I, II, III and IV 1, 2. A series of studies have discovered that anti‐diabetic agents like metformin and berberine inhibited the function of mitochondrial complex I 3, 4, 5, 6. In our previous studies, berberine decreased oxygen consumption and increased AMP/ATP ratio, two indicators of mitochondrial function, in hepatocytes, myotubes and adipocytes 7, 8. We further performed extracellular flux analysis and found complex I—linked respiration was almost abolished in C2C12 myotubes treated with metformin or berberine 9. Therefore, we hypothesized that complex I may be a drug target for these glucose‐lowering agents. To test the hypothesis, db/db mice, a diabetic model caused by genetic defects, and chemical inhibitors, for example rotenone and amobarbital, and gene silencing of NDUFA13*,* a component of complex I, were used in this study. We discovered complex I inhibition alleviated hyperglycaemia through induced glycolysis and reduced hepatic glucose output.

Adenosine monophosphate‐activated protein kinase (AMPK), a highly conserved sensor of cellular energy status, exists in almost all eukaryotes. Phosphorylation of Thr‐172 is used as a biomarker of AMPK activation 10. Since 2001, AMPK has been widely considered as molecular effecter of metformin when Zhou *et al*. 11 reported that metformin stimulated AMPK, which was associated with inhibition of glucose production in rat primary hepatocytes. Furthermore, Shaw *et al*. 12 proposed LKB1‐AMPK pathway played a central role in mechanisms of metformin action. Strikingly, several laboratories including us found that berberine was able to activate AMPK, too 7, 13, 14. However, the key role of AMPK in the effects of metformin and berberine has been challenged recently. Foretz *et al*. 15 reported that metformin‐induced inhibition of glucose production was even greater in both AMPK‐ and LKB1‐deficient hepatocytes than in wild‐type cells. In our previous work, when AMPK was inactivated, both berberine and metformin could still stimulate glycolysis through suppressing respiratory chain at complex I 9. Thus, whether AMPK or complex I is the target of metformin and berberine remains in conflict. To address this issue and further illuminate the role of AMPK in the beneficial effects of complex I inhibition, we tested the alteration of glucose homeostasis by rotenone in the absence of AMPK activation in this study. Suppression of hepatic glucose output and enhancement of glycolysis with rotenone were still observed without AMPK activation. Besides, we found rotenone decreased cellular nicotinamide adenine dinucleotide (NAD^+^)/reduced form of nicotinamide‐adenine dinucleotide (NADH) ratio, which may be related to the enhanced glycolysis and diminished gluconeogenesis by complex I inhibition.

## Materials and methods

### Animal studies

Eight‐week‐old male db/db (+/+) C57/BL/KsJ mice (25 ± 3 g) were purchased from Experimental Animals Center of Chinese Academic (Shanghai, China) and housed in animal facility at (22 ± 3) °C with a 12‐hrs light/dark cycle. The mice were housed at three to four per cage with free access to water and diet. The animals were randomly divided into control (*n* = 6) and rotenone‐treated (*n* = 6) groups. Before treatment, FBG was measured by the glucose oxidase method (Roche, Switzerland) and recorded. Daily food intake and weight change of each mouse were continuously counted and recorded. Rotenone (1 mg/kg) was intraperitoneally injected once daily for 2 weeks. An equal volume of vehicle (distilled water and 95% ethylalcohol mixture) was delivered in control mice. After 2‐week rotenone treatment, intraperitoneal glucose tolerance test (IPGTT) was carried out with intraperitoneal injection of 0.1 g/kg glucose after overnight fasting. Blood glucose concentrations were assessed before (0 min.) and 15, 30, 60 and 120 min. after glucose injection. Insulin tolerance test (ITT) was conducted by intraperitoneal injection of 1 unit/kg regular insulin (Novo Nordisk, Denmark) to fasted mice, and blood glucose levels were measured at 0, 15, 30, 60, and 120 min. Fasting insulin levels (FINS) were determined using ELISA kit (Sino Biological Inc. China). All procedures involving the care and use of animals were performed according to Shanghai Jiao Tong University Guidelines for the care and use of laboratory animals.

### Cell culture

The HepG2 hepatocytes and C2C12 mouse skeletal myoblasts were obtained from Shanghai Diabetes Institute. They were grown in DMEM (Invitrogen, NY, USA) containing 10% fetal bovine serum (FBS), 100 units/ml penicillin and 0.1 mg/ml streptomycin in an incubator of 5% CO_2_ at 37°C (low‐glucose DMEM for HepG2 cells and high‐glucose DMEM for C2C12 cells). For differentiation of myotubes, C2C12 cells were seeded into 12‐well plates in DMEM containing 10% FBS for 24 hrs. Then, the medium was replaced by DMEM with 2% horse serum, 100 units/ml penicillin and 0.1 mg/ml streptomycin for every 2 days. After 6 days, the C2C12 myotubes were ready for experiments.

### Glucose consumption

Before the experiments, the cells were transferred and maintained in 96‐well plates to 80% confluence and then treated with rotenone or amobarbital at various concentrations in FBS‐free DMEM (15 mmol/l D‐glucose) containing 0.25% bovine serum albumin (BSA) for 24 h (*n* = 4–8/group). The glucose concentration in the medium was determined by the glucose oxidase method (Applygen Technologies Inc., China). The amount of glucose consumption was calculated by subtraction of glucose concentrations between the cell plated wells and the blank wells 7. The cells were collected and lysed. And the total protein amount of each well was measured using a BCA protein assay kit (Beyotime Biotechonology, China) to normalize the glucose consumption.

### Lactate release

Cells were grown to 80% confluence in 96‐well plates and added with rotenone or amobarbital in DMEM supplemented with 0.25% BSA for 24 hrs (*n* = 4–8/group). The lactate concentration in the medium was determined with a lactic acid assay kit (Shanghai Juchuang Biotechnology Corporation, China). The amount of lactate release was calculated by subtraction of lactate concentrations between the cell plated wells and the blank wells.

### Primary hepatocytes isolation

Primary hepatocytes were isolated from C57BL/6 mice by a previously described two‐step perfusion method with some modifications 16, 17, and seeded on six‐well, gelatine‐coated plates in RPMI1640 medium (Invitrogen) containing 10% FBS and 100 U/ml antibiotic/antimycotic. The viability of the cell preparation was at least 85% (as determined by trypan blue exclusion prior to plating cells). After 4–6 hrs of attachment, the medium was replaced with fresh media.

### Glucose output assay

Primary hepatocytes were isolated and plated in RPMI1640 medium with FBS and antibiotic/antimycotic solution for 4–6 hrs. After attachment, the medium was replaced with serum‐free low‐glucose DMEM medium. After 12 hrs, the cells were washed with phosphate‐buffered saline (PBS) three times and then incubated in 1 ml/well of phenol red‐free, glucose‐free DMEM supplemented with gluconeogenic substrates (20 mM lactate, 2 mM pyruvate, 10 mM glutamine) and different concentrations of rotenone (Sigma‐Aldrich) or amobarbital (Langchem Inc. China) for 8 hrs (*n* = 4–8/group). Medium 50 μl was taken to measure the glucose concentration in the culture medium using a glucose assay kit (Applygen Technologies Inc., China). A twofold concentration of the kit reagents was used to increase the sensitivity. The cells were collected and lysed. And the total protein amount of each well was measured using a BCA protein assay kit (Beyotime Biotechonology, China) to normalize the glucose output.

### LDH cytotoxicity assay

Cells were cultured to 80% confluence in 96‐well plates. After rotenone treatment for 24 hrs in serum‐free DMEM supplemented with 0.25% BSA, lactate dehydragenase contents in the medium and cells were detected with the Lactate Dehydrogenase (LDH)—Cytotoxicity Assay Kit II (Beyotime Biotechonology, China) (*n* = 3–8/group). The percentage of living cells was calculated by the following equation: living cells (%) = 100 – (OD_490_treated – OD_490_control)/(OD_490_maximum enzyme activity – OD_490_control) × 100.

### NAD^+^/NADH ratio assay

Cells were treated with different concentrations of rotenone for 2 hrs (*n* = 3–6/group). Wash cells with cold PBS and pellet ~10^5^ cells for each sample. Homogenize samples with 100 μl NAD^+^ or NADH extraction buffer (BioAssay Systems, CA, USA). The assay was conducted following the manufacturer's instructions.

### Liver mitochondria isolation and oxidative phosphorylation analysis

Intact mitochondria were isolated as previously described 18. Mice were fasted overnight before the experiment. Mitochondrial concentration was measured using the Bradford protein assay kit (Beyotime Biotechonology, China). For oxidative phosphorylation analysis, isolated mitochondria were conducted at 37°C in a Clark type oxygen electrode (Strathkelvin Instruments, Scotland). The respiration medium consisted of 225 mM mannitol, 75 mM sucrose, 10 mM Tris–HCl, 10 mM KH_2_PO_4_, 10 mM KCl, 0.8 mM MgCl_2_, 0.1 mM EDTA, 0.3% fatty acid free BSA, pH 7.0. Isolated mitochondria were transferred to the electrode chamber and allowed to equilibrate until they attained a steady rate of oxygen consumption. Rotenone was added dose dependently, and their effect on oxygen consumption was recorded (*n* = 3–6/group). Dose–response effects of rotenone on mitochondrial respiration were determined in the presence of 0.2 mM ADP, using substrates in combination (5 mM pyruvate plus 2 mM malate) targeting complex I of the respiratory chain.

### Adenoviruses infection

Recombinant adenoviruses expressing dominant negative forms of AMPKα1 (D159A) and AMPKα2 (K45R) were purchased from Asia Biological Science Technologies Co., Shanghai. The α1/α2‐DN was used to block AMPK activity as previously described 19, 20. Cells were infected with adenoviruses expressing control GFP reporter protein or dominant site mutagenesis AMPKα1/α2 (DN‐AMPK) for 5–6 hrs and treated with rotenone after infection (*n* = 3–8/group).

### RNA interference

HepG2 cells were transfected with siRNA directed against *NDUFA13* or scramble siRNA (Santa Cruz, USA) and corresponding 1/20 concentration of Lipofectamine 2000 Transfection Reagent (Invitrogen) for 24 hrs. The delivery of scramble or *NDUFA13* siRNA into primary hepatocyte cells was performed by an Amaxa‐based electroporation method (Amaxa). RNAi (10 pmol) was used to transfect 7 × 10^5^ cells in each electroporation.

### Western blot

After treatment with different concentrations of rotenone for 24 hrs in HepG2 cells and C2C12 cells or 8 hrs in primary hepatocytes, cells were lysed using RIPA buffer containing 1 mM PMSF and phosphatase inhibitor cocktail. The lysates (35 μg) were boiled for 5 min. and separated by SDS‐polyacrylamide gel electrophoresis (SDS‐PAGE). Then, the separated proteins were transferred onto polyvinylidene fluoride (PVDF) membrane (Bio‐Rad). After blocked with 5% skim milk in the Tris‐Buffered Saline Tween‐20 (TBST) buffer for 1 hr at room temperature, the membrane was probed overnight at 4°C with primary antibody (AMPKα: 1:1000, Cell Signaling Technology; phospho‐AMPKα: 1:1000, Cell Signaling Technology; ACC: 1:1000, Cell Signaling Technology; phospho‐ACC:1:1000, Cell Signaling Technology; GRIM‐19: 1:200, Santa Cruz; β‐actin: 1:1000, Cell Signaling Technology; GAPDH: 1:1000, Cell Signaling Technology) and then washed and re‐blotted with HRP conjugated secondary antibody for 1 hr. All secondary antibodies were from Cell Signaling Technology and used at 1:500–1000 dilution. Immunoreactive bands were visualized with ECL reagent (Thermo Fisher Scientific Inc., USA). Gel‐Pro Analyzer 4.0 was used to quantify the Western signals.

### Statistical analysis

Data are expressed as means ± SEM. All experiments were performed a minimum of three times. Two‐tailed Student's *t*‐test, one‐way anova and two‐way anova with Bonferroni's correction (SPSS 20.0) were used in statistical analysis. A level of *P* < 0.05 was considered statistically significant.

## Results

### Rotenone decreased blood glucose and ameliorated insulin resistance in db/db mice

**Figure 1:**
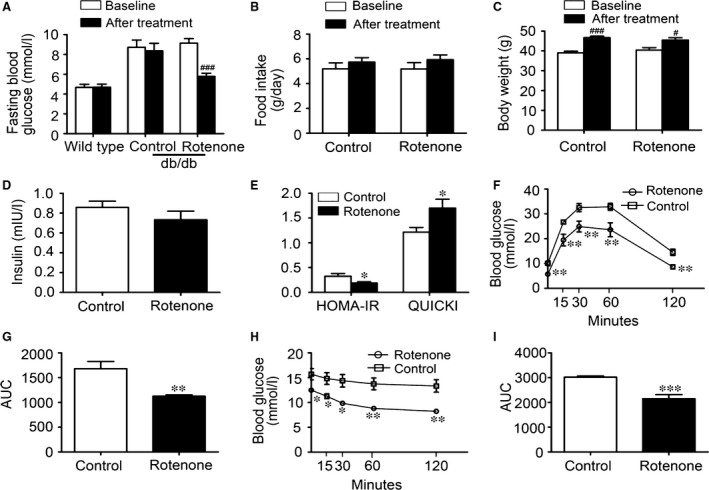
Rotenone improved glucose homoeostasis and insulin sensitivity in db/db mice. (**A**) fasting blood glucose levels, (**B**) food intake, (**C**) body weight, (**D**) fasting serum insulin concentrations, (**E**) HOMA‐IR and QUICKI, (**F**) OGTT and (**H**) ITT curves, (**G** and **I**) AUC of OGTT and ITT from two‐week rotenone or vehicle treated mice. Data were expressed as means ± SEM (*n* = 6). ^#^ *P* < 0.05, ^###^ *P* < 0.001 *vs*. corresponding baseline. *P < 0.05, **P < 0.01, ***P < 0.001 *vs*. db/db mice treated with vehicle.

Firstly, we assessed the change of glucose homoeostasis and insulin sensitivity in diabetic db/db mice treated with rotenone (*n* = 6/group). The mean baseline weight of db/db mice in control group and rotenone treated group were (39.0 ± 2.1) g and (40.4 ± 3.1) g, respectively. After rotenone 1 mg/kg administration for 2 weeks, fasting blood glucose (FBG) and fasting insulin levels were measured. IPGTT and ITT were conducted, too. Compared to control db/db mice, rotenone‐treated db/db mice exhibited remarkably reduced FBG levels (Fig. 1A). No differences in body weight and daily food intake were noted between the two groups (Fig. 1B and C). Fasting serum insulin levels were a little bit lower after rotenone treatment, although the difference did not reach statistical significance (Fig. 1D). HOMA‐IR and QUICKI were used to assess insulin sensitivity of the mice. Rotenone‐treated db/db mice showed markedly decreased HOMA‐IR and significantly increased QUICKI (Fig. 1E), implying improved insulin sensitivity. Furthermore, blood glucose levels and area under curve (AUC) of rotenone‐treated db/db mice were significantly lower during IPGTT (Fig. 1F and G), suggesting an improvement in systemic glucose tolerance. Similarly, rotenone treatment led to substantially decreased blood glucose levels and AUC during ITT (Fig. 1H and I), indicating enhanced insulin response.

### Rotenone lowered glucose by promoting glycolysis and inhibiting hepatic glucose output *in vitro*

**Figure 2:**
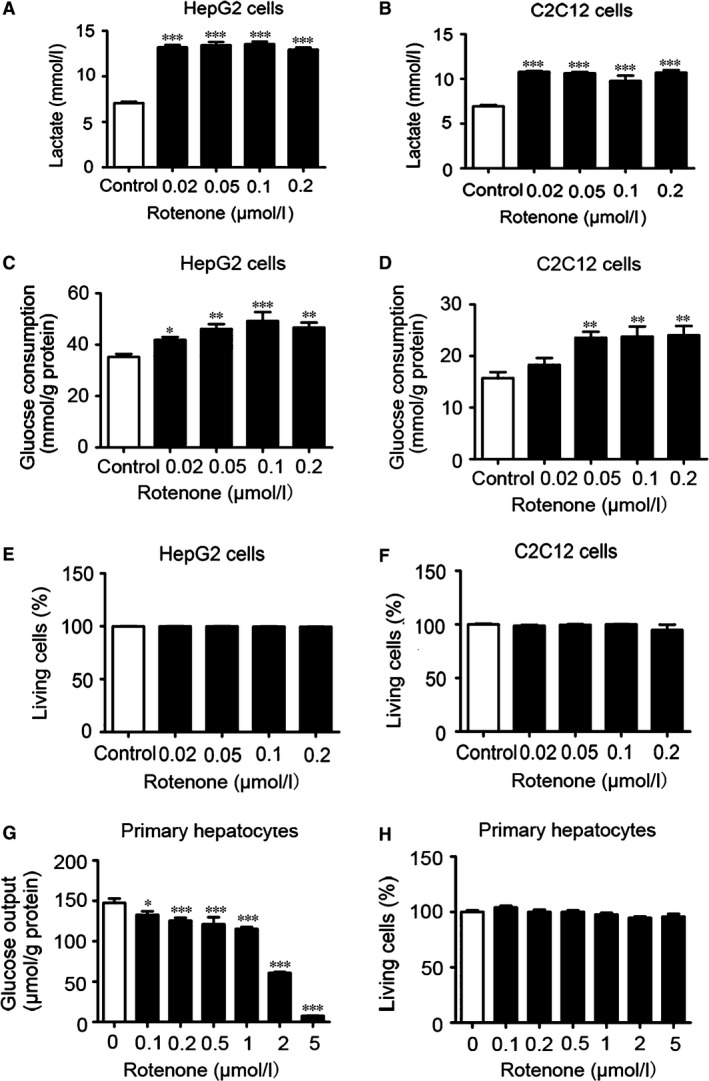
Rotenone promoted glycolysis and inhibited gluconeogenesis without cytotoxicity in cells. After incubation with rotenone for 24 hrs, lactate production, glucose consumption and LDH concentration were measured in (**A, C** and **E**) HepG2 hepatocytes and (**B, D** and **F**) C2C12 myotubes. (**G**) Glucose output and (**H**) LDH concentrations in primary hepatocytes were measured after rotenone treatment for 8 hrs. Data are expressed as means ± SEM. (*n* = 4–8); *P < 0.05, **P < 0.01 and ***P < 0.001 *vs*. corresponding control.

To explore the hypoglycaemic effect of rotenone, anaerobic respiration indicated by lactate release in HepG2 hepatocytes and C2C12 myotubes was also measured (*n* = 4–8/group). A 1.8‐fold induction in lactate production of HepG2 cells was observed after rotenone treatment for 24 hrs (Fig. 2A). Similar effect was shown in C2C12 cells, too (Fig. 2B). These data demonstrated that rotenone may induce glycolysis *via* inhibition of complex I *in vitro*. Additionally, at concentrations between 0.02 and 0.2 μmol/l, rotenone promoted glucose consumption of HepG2 cells by 19.0–32.7% (Fig. 2C) in a dose‐dependent manner. Similar effects were observed in C2C12 cells (Fig. 2D). As rotenone impaired mitochondrial capacity at complex I, it was necessary to ascertain whether this agent had toxicity in the cells. Therefore, LDH cytotoxicity assay was performed to address this issue. Exposure to rotenone up to 24 hrs with various concentrations used in our *in vitro* study did not alter LDH release in HepG2 hepatocytes (Fig. 2E). Similar results were detected in C2C12 myotubes (Fig. 2F). As enhanced gluconeogenesis played an important role in hyperglycaemia of diabetes, we further explored the effect of rotenone on hepatic glucose output. Treatment with rotenone for 8 hrs strongly inhibited glucose production in primary mouse hepatocytes (Fig. 2G). In addition, LDH release did not change in primary mouse hepatocytes exposed to rotenone up to 8 hrs with various concentrations (Fig. 2H). Rotenone may improve glucose metabolism through inducing glycolysis and reducing hepatic gluconeogenesis.

### Rotenone stimulated AMPK pathway

**Figure 3:**
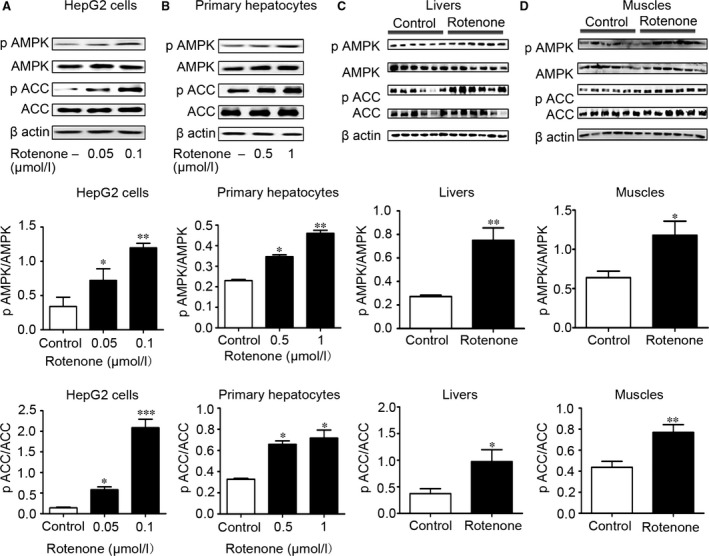
Rotenone stimulated AMPK and ACC phosphorylation *in vitro* and *in vivo*. (**A–D**) Representative Western blot for AMPK, ACC, phospho‐AMPKα (Thr172) and phospho‐ACC (Ser79) in HepG2 hepatocytes after rotenone treatment for 24 hrs (**A**), in primary hepatocytes after rotenone treatment for 8 hrs (**B**), or in livers (**C**) and muscles (**D**) from db/db mice after rotenone treatment for 2 weeks. Data are expressed as means ± SEM. (*n* = 3–6); *P < 0.05, **P < 0.01, ***P < 0.001 *vs*. control.

To clarify the relationship between mitochondrial inhibition and stimulation of AMPK pathway, we assessed the effect of rotenone on AMPK activity (*n* = 3–6/group). In HepG2 cells, up to 2.5‐ and 14‐fold induction of the phosphorylation levels of AMPK (Thr_172_) and its downstream target protein acetyl‐CoA carboxylase (ACC) (Ser_79_) were observed after rotenone treatment for 24 hrs (Fig. 3A). Significant increase in AMPK and ACC phosphorylation was also observed in primary hepatocytes after incubation with rotenone for 8 hrs (Fig. 3B). Liver and muscle of the db/db mice were collected for immunoblotting. Rotenone treatment for 2 weeks augmented phosphorylation level of AMPK and ACC in the livers by 2.8‐ and 2.6‐fold compared with control db/db mice (Fig. 3C). Meanwhile, in the muscles, rotenone resulted in 1.8‐ and 1.7‐fold increase of AMPK and ACC phosphorylation, respectively (Fig. 3D). All these results suggested that rotenone was able to induce AMPK phosphorylation through suppressing mitochondrial complex I.

### Rotenone‐stimulated glycolysis was not blocked in the absence of AMPK activation

**Figure 4:**
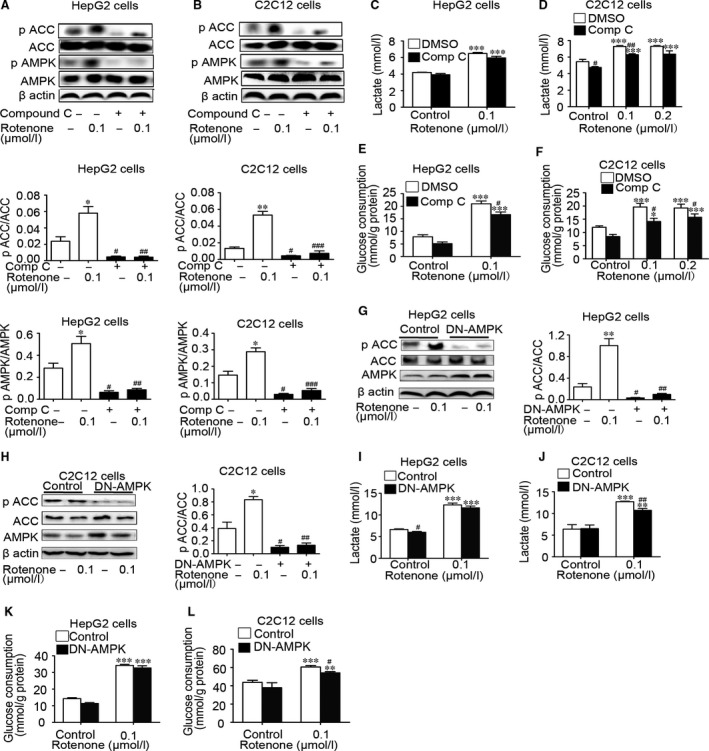
Rotenone enhanced glycolysis in the absence of AMPK activation. Representative Western blot for AMPK, ACC, phospho‐AMPKα (Thr172) and phospho‐ACC (Ser79) in HepG2 cells (**A**) and C2C12 cells (**B**) pre‐incubated with compound C or DMSO for 30 min. Lactate release and glucose consumption were detected in HepG2 hepatocytes (**C** and **E**) and C2C12 myotubes (**D** and **F**). Western blot for AMPK, ACC and phospho‐ACC (Ser79) in HepG2 cells (**G**) and C2C12 cells (**H**) infected with adenoviruses expressing control GFP reporter protein or DN‐AMPK for 5–6 hrs. Lactate release and glucose consumption were determined in HepG2 (**I** and **K**) and C2C12 (**J** and **L**) cells. Data are expressed as means ± SEM (*n* = 3–8). *P < 0.05, **P < 0.01, ***P < 0.001 *vs*. the cells without rotenone treatment; ^#^ *P* < 0.05, ^##^ *P* < 0.01, ^###^ *P* < 0.001 *vs*. the cells without compound C treatment or DN‐AMPK infection.

To determine whether AMPK activation was involved in the anti‐diabetic effect of rotenone, HepG2 hepatocytes and C2C12 myotubes were pre‐incubated with 10 μmol/l compound C, an AMPK inhibitor, or vehicle (DMSO) for 30 min., followed by rotenone treatment for 24 hrs (*n* = 3–8/group). As shown in Fig. 4A and B, compound C markedly decreased rotenone‐induced AMPK and ACC phosphorylation, suggesting it had inhibited AMPK activity in HepG2 cells and C2C12 cells. With treatment of compound C, rotenone still promoted lactate release (Fig. 4C and D) and glucose consumption (Fig. 4E and F) in both cell lines. Interestingly, the levels of glucose expenditure and lactate release in compound C‐treated cells were modestly below the corresponding control, indicating AMPK inhibition may slightly interfere glucose metabolism whatever rotenone presented or not. The increasing rates of lactate release and glucose consumption stimulated by rotenone had no significant differences between control and compound C groups (Fig. S1A and B). To ulteriorly prove that glucose‐lowering action of rotenone did not depend on AMPK, the DN‐AMPK adenoviruses were employed to inhibit AMPK pathway. As shown in Fig. 4G and H, compared with control GFP reporter protein adenovirus, DN‐AMPK adenovirus expressed more AMPKα in HepG2 hepatocytes and C2C12 myotubes, while rotenone‐induced ACC phosphorylation was completely reversed. However, the stimulated lactate release (Fig. 4I and J) and glucose consumption (Fig. 4K and L) by rotenone was not altered. If ratio of rotenone treatment to control was compared, DN‐AMPK adenoviruses even increased rotenone‐induced glucose consumption by 12.3% in HepG2 cells (Fig. S1C and E). In C2C12 myotubes, the ratio of rotenone treatment to control in glucose utilization did not alter with DN‐AMPK adenoviruses infection, although that of lactate release decreased from 2.0‐ to 1.7‐fold (Fig. S1D and F). These results demonstrated that rotenone promoted glycolysis independently of AMPK pathway.

### Rotenone suppressed hepatic gluconeogenesis in the absence of AMPK activation

**Figure 5:**
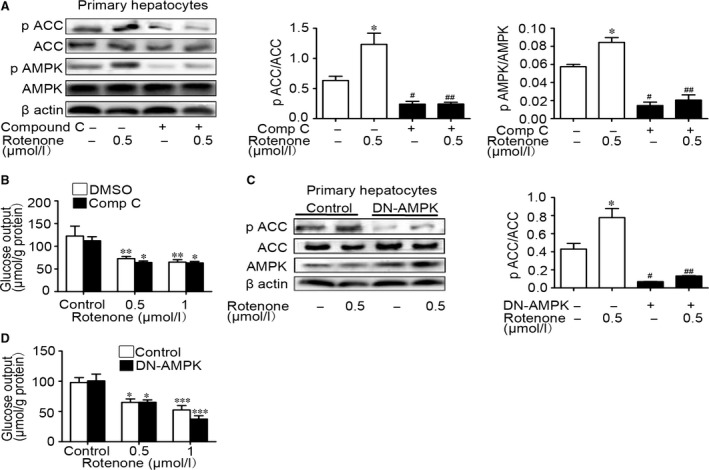
Rotenone inhibited hepatic glucose output in the absence of AMPK activation. The protein level of AMPK, ACC, phospho‐AMPKα (Thr172) and phospho‐ACC (Ser79) was examined by Western blot (**A**) in primary hepatocytes pre‐incubated with compound C or DMSO for 60 min. Glucose output was measured (**B**). The protein level of ACC phosphorylation was examined by Western blot (**C**) in primary hepatocytes infected with adenoviruses expressing control GFP reporter protein or DN‐AMPK for 5–6 hrs. Glucose output was measured in the hepatocytes (**D**). Data are expressed as means ± SEM (*n* = 3–8). *P < 0.05, **P < 0.01, ***P < 0.001 *vs*. the cells without rotenone treatment. ^#^ *P* < 0.05, ^##^ *P* < 0.01 *vs*. the cells without compound C treatment.

In primary hepatocytes, pretreatment of compound C for 1 hr markedly decreased rotenone‐induced AMPK and ACC phosphorylation, but rotenone still restrained hepatic gluconeogenesis by up to 43.5% (Fig. 5A and B) (*n* = 3–8/group). That suggested compound C did not influence rotenone‐reduced glucose output. As shown in Fig. 5C, DN‐AMPK adenoviruses blocked ACC phosphorylation in the primary hepatocytes, suggesting AMPK activity was inhibited. Inhibitory effect of rotenone (0.5 μmol/l) on glucose output was not repealed in the primary hepatocytes infected with DN‐AMPK adenoviruses, which even enhanced the inhibition of rotenone at 1 μmol/l on glucose output (Fig. 5D). To sum up, these data indicated that AMPK may be dispensable for suppressed hepatic glucose output by rotenone.

### Rotenone decreased cellular NAD^+^/NADH ratio and inhibited complex I—linked respiration

**Figure 6:**
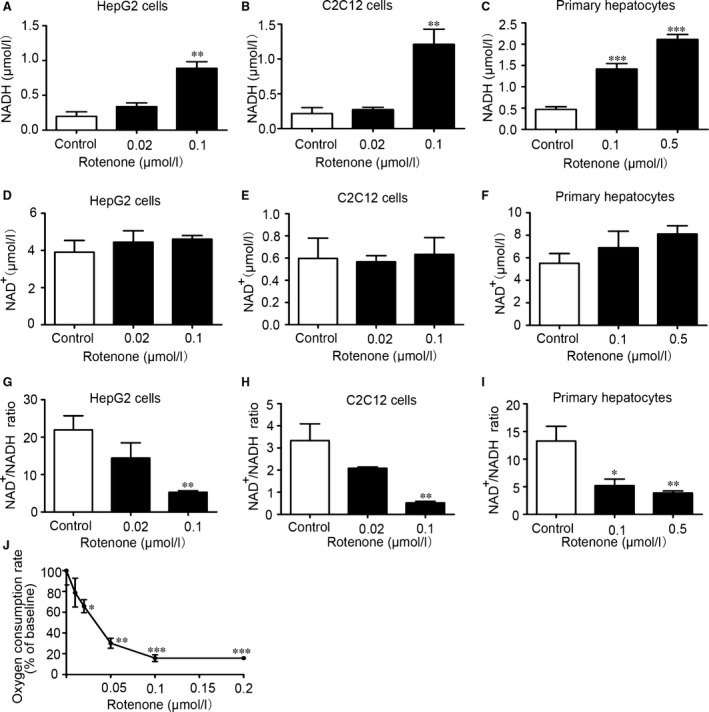
Rotenone increased cellular NADH content, decreased cellular NAD ^+^/NADH ratio and inhibited complex I—linked respiration. NADH levels, NAD ^+^ levels and NAD ^+^/NADH ratios were determined in HepG2 hepatocytes (**A, D** and **G**), C2C12 myotubes (**B, E** and **H**) and primary hepatocytes (**C, F** and **I**) after rotenone treatment for 2 hrs. (**J**) The liver mitochondrial oxygen consumption rate was measured after rotenone treatment. Data are expressed as means ± SEM (*n* = 3–6). *P < 0.05, **P < 0.01, ***P < 0.001 *vs*. control.

To explore the effect of rotenone on mitochondrial respiratory chain complex I, cellular NAD^+^ and NADH concentrations were measured after rotenone treatment for 2 hrs (n = 3–6/group). In HepG2 cells and C2C12 cells, NADH concentrations were markedly increased after treatment with 0.1 μmol/l rotenone (Fig. 6A and B). In primary hepatocytes, rotenone at 0.1 and 0.5 μmol/l significantly increased NADH concentrations (Fig. 6C). However, rotenone did not alter NAD^+^ concentrations in the three cell models (Fig. 6D, E and F). We further calculated and compared NAD^+^/NADH ratios. NAD^+^/NADH ratios in HepG2 cells showed a downward trend after 2‐hrs treatment with 0.02 μmol/l rotenone. Rotenone at 0.1 μmol/l significantly decreased NAD^+^/NADH ratios compared with control group (Fig. 6G). Similar effects were observed in C2C12 cells (Fig. 6H). In primary hepatocytes, NAD^+^/NADH ratios were significantly decreased after 2‐hrs incubation with 0.1 and 0.5 μmol/l rotenone (Fig. 6I). Additionally, oxygen consumption rates (OCR) were measured in the presence of physiological dose of ADP, using complex I‐targeted substrate combinations (pyruvate plus malate). As shown in Fig. 6J, at concentrations between 0.02 and 0.2 μmol/l, rotenone immediately and strongly decreased OCR in isolated liver mitochondria. These data indicated that rotenone disrupted respiratory chain complex I, resulting in reduction of NAD^+^/NADH ratio and OCR.

### Complex I inhibition by amobarbital treatment and NDUFA13 silencing promoted glucose metabolism *in vitro*

**Figure 7:**
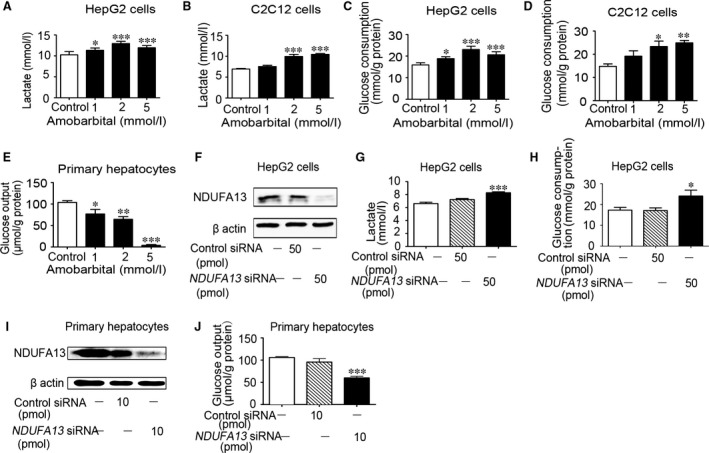
Amobarbital and silencing of *NDUFA13* promoted glucose metabolism *in vitro*. Lactate release and glucose consumption were detected in HepG2 (**A** and **C**) and C2C12 (**B** and **D**) cells after amobarbital incubation for 24 hrs. (**E**) Glucose output of primary hepatocytes with amobarbital treatment for 8 hrs. Extracts from the cells were measured by Western blot for NDUFA13 (**F**) in HepG2 cells transfected with control or *NDUFA13* siRNA for 24 hrs. Lactate release (**G**) and glucose consumption (**H**) were detected. (**I**) Western blot for Grim19 in primary hepatocytes transfected with control or *NDUFA13* siRNA for 24 hrs. (**J**) Glucose output in the primary hepatocytes. Data are expressed as means ± SEM. (*n* = 6–8); *P < 0.05, **P < 0.01, ***P < 0.001 *vs*. control.

To test whether other mitochondrial complex I inhibitors have the similar effects as rotenone, amobarbital, another established complex I inhibitor, was also applied (*n* = 6–8/group). Amobarbital treatment for 24 hrs facilitated lactate release both in HepG2 and C2C12 cells (Fig. 7A and B). Glucose consumption in amobarbital‐treated group was significantly elevated, too (Fig. 7C and D). As in primary cultured hepatocytes, glucose output was remarkably depressed by amobarbital treatment for 8 hrs (Fig. 7E). Gene silencing of NADH dehydrogenase [ubiquinone] 1 α subcomplex subunit 13 (NDUFA13), a component of complex I, is able to hinder the function of mitochondrial complex I. In HepG2 hepatocytes, *NDUFA13* siRNA (50 pmol/well) notably decreased the expression of *NDUFA13* in comparison with scramble siRNA (Fig. 7F). After 24‐hrs transfection of *NDUFA13* siRNA, the glucose consumption and lactate production were promoted by 39.3% and 25.2%, respectively (Fig. 7G and H). We also transfected *NDUFA13* siRNA (10 pmol/well) into primary hepatocytes using electroporation (Fig. 7I) for 24 hrs and found the glucose output was obviously reduced (Fig. 7J). These results confirmed restraining the activity of complex I could not only accelerate glycolysis and glucose expenditure in HepG2 and C2C12 cells, but also suppress gluconeogenesis in primary cultured hepatocytes.

## Discussion

Mitochondria are intracellular organelles which devote mainly to cellular energy production *via* the oxidative phosphorylation system 1, 2. Several classical antihyperglycaemic drugs such as metformin, berberine and thiazolidinediones were reported to exert glucose‐lowering effect through repression of complex I. Thus, we explored the potential therapeutic effect of inhibiting mitochondria at complex I in treatment of hyperglycaemia. Two‐week administration of rotenone effectively lowered FBG, improved impaired glucose tolerance and insulin sensitivity of db/db mice. For the first time, we demonstrated the complex I inhibition by rotenone had glucose lowering effect on the diabetic animals.

We further investigated the beneficial metabolic action of complex I inhibition in hepatocytes and myotubes. A parallel elevation in lactate release and glucose utilization was observed in HepG2 and C2C12 cells after rotenone treatment. The lactate in the culture medium is produced *via* glycolysis process, which occurs outside mitochondria and transfers energy from glucose to ATP without consuming oxygen. Glycolysis is less efficient in ATP synthesis and requires more glucose in the production of the same amount of ATP than aerobic respiration 7. Thus, glycolysis may lead to increased glucose consumption in hepatic and muscular cells. In addition, rotenone inhibited gluconeogenesis in primary mouse hepatocytes. These results may explain rotenone‐elicited decrease of blood glucose in db/db mice.

NDUFA13, a protein with a specific role in cell death regulation, is indispensable for assembly and function of mitochondrial complex I. Demolition of NDUFA13 destroys the assembly and electron transfer activity of complex I 21. Thus, we used *NDUFA13* siRNA to further investigate the impact of suppressing complex I on metabolic alteration. Reduction in NDUFA13 *via* siRNA exerted similar effects as rotenone on glucose metabolism. Moreover, amobarbital, a well‐known reversible complex I inhibitor, was also deployed in this study. Amobarbital showed almost identical action in stimulating glycolysis and reducing hepatic glucose output as NDUFA13 down‐regulation. To sum up, this work was the first to study glucose metabolism *via* repression of complex I by two means, chemical blockers and post‐transcriptional gene silencing. Both methods resulted in improved glucose homoeostasis. It is reasonable to speculate that complex I inhibition may be the crucial point to remedy the disorder of glucose metabolism.

This study showed rotenone stimulated phosphorylation of AMPK and ACC in a dose‐dependent manner *in vitro*. Additionally, phosphorylation of AMPK and ACC in liver and muscle of the db/db mice was also induced after rotenone treatment. That indicated complex I inhibition resulted in AMPK activation. The mechanism suggests that AMPK activation by metformin and berberine may be the consequence of the agents’ complex I inhibition, too 22. However, whether AMPK was involved in the glucose lowering effect of complex I inhibition remained to be addressed. Thus, we further tested action of rotenone in the absence of AMPK activation. Our *in vitro* data showed that rotenone induced glycolysis and reduced hepatic glucose output when AMPK was inactivated with compound C or DN‐AMPK adenoviruses. These results are consistent with our previous study that metformin and berberine were able to stimulate glucose consumption and lactate release with AMPK being inactivated 9, despite both of them are well‐known as AMPK activators. Other groups also reported that metformin repressed hepatic gluconeogenesis and glucagon signalling independently of AMPK pathway 15, 23. This study demonstrated that complex I inhibition was able to improve glucose metabolism directly regardless of AMPK activity.

**Figure 8:**
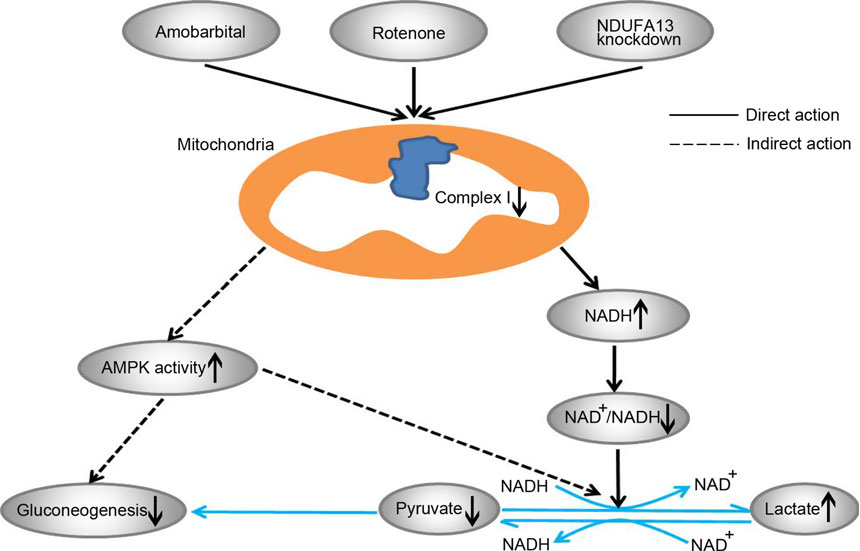
Proposed mechanisms of mitochondrial complex I inhibition in regulating glucose metabolism. Rotenone, amobarbital or NDUFA13 knockdown inhibits the activity of oxidative respiratory chain complex I. This leads to an increase in cellular NADH content and reduction in NAD ^+^/NADH ratio. Elevated NADH level facilitates pyruvate into glycolytic process ratio. And gluconeogenic pathway is repressed due to lack of pyruvate. On the other hand, AMPK restrains gluconeogenic flux and stimulates the conversion of pyruvate to lactate in an indirect way.

In our research, rotenone was found to increase NADH level and decrease cellular NAD^+^/NADH ratio. It is known that pyruvate is at the nexus of glycolysis and gluconeogenesis. NADH is oxidized to NAD^+^ in the electron transport chain complex I. We speculated that in response to mitochondrial complex I dysfunction, NADH levels are elevated, promoting the conversion of pyruvate to lactate, concomitantly converting NADH to NAD^+^. Decreased NAD^+^/NADH ratio facilitated pyruvate into glycolytic pathway. On the other hand, gluconeogenic pathway was repressed due to reduced pyruvate. These findings revealed that the antihyperglycaemic effect of complex I inhibition may be associated with suppression of cellular NAD^+^/NADH ratio (Fig. 8).

In this study, inhibition of complex I presented no toxicity in all experiments. In addition to the beneficial effects of complex I inhibition on glucose metabolism, a number of studies revealed that administration of complex I inhibitor, that is rotenone or amobarbital, before cardiac ischaemia onset significantly attenuated damage to mitochondria and decreased myocardial injury 24, 25, 26, 27, 28, 29. The UK Prospective Diabetes Study (UKPDS) Group reported metformin significantly reduced cardiovascular mortality in overweight diabetic patients 30, the protective effect of metformin on cardiovascular system may be due to the complex I inhibition. As the majority of diabetic patients die of macrovascular complications, drug companies are always struggling to develop novel antihyperglycaemic agents with protective action on cardiovascular diseases. Complex I inhibition may be a promising direction for the future antidiabetic drugs.

In conclusion, our results demonstrate glucose metabolism improves as long as complex I activity is repressed regardless of whether chemical reagents or gene silencing is used. Complex I inhibition is able to alleviate hyperglycaemia in diabetic mice *via* inducing glycolysis and reducing hepatic glucose output. Furthermore, AMPK activation is not indispensable to the action of complex I inhibition. The antihyperglycaemic effect of complex I inhibition may be related to the suppression of cellular NAD^+^/NADH ratio. Our research provides evidences that mitochondrial complex I is an emerging drug target for diabetes.

## Disclosures

The authors have no financial disclosures relevant to the material described in this manuscript.

## Author contributions

J.Y. is the guarantor of this work and, as such, had full access to all the data in the study and takes responsibility for the integrity of the data and the accuracy of the data analysis. J.Y. and F.L. designed the studies. WL.H. carried out the research. XY.Y, M.A and LG.A assisted in performing research. WL.H. and J.Y. interpreted the results and wrote the manuscript. F.L., YQ.B. and WP.J. assisted in reviewing and revising the manuscript. All authors approved the final version of the manuscript.

## Supporting information


**Figure S1** The increasing rates of lactate release and glucose consumption stimulated by rotenone did not change with AMPK inactivation.
