# Supplementary material for: Inhibition of mitochondrial complex I improves glucose metabolism independently of AMPK activation
Source: J Cell Mol Med. 2017 Nov 6;22(2):1316–28. doi: 10.1111/jcmm.13432 (PMC5783883; doi:10.1111/jcmm.13432)
Supplement: Supplementary file 1 — Figure S1 The increasing rates of lactate release and glucose consumption stimulated by rotenone did not change with AMPK inactivation. [file JCMM-22-1316-s001.docx]

**
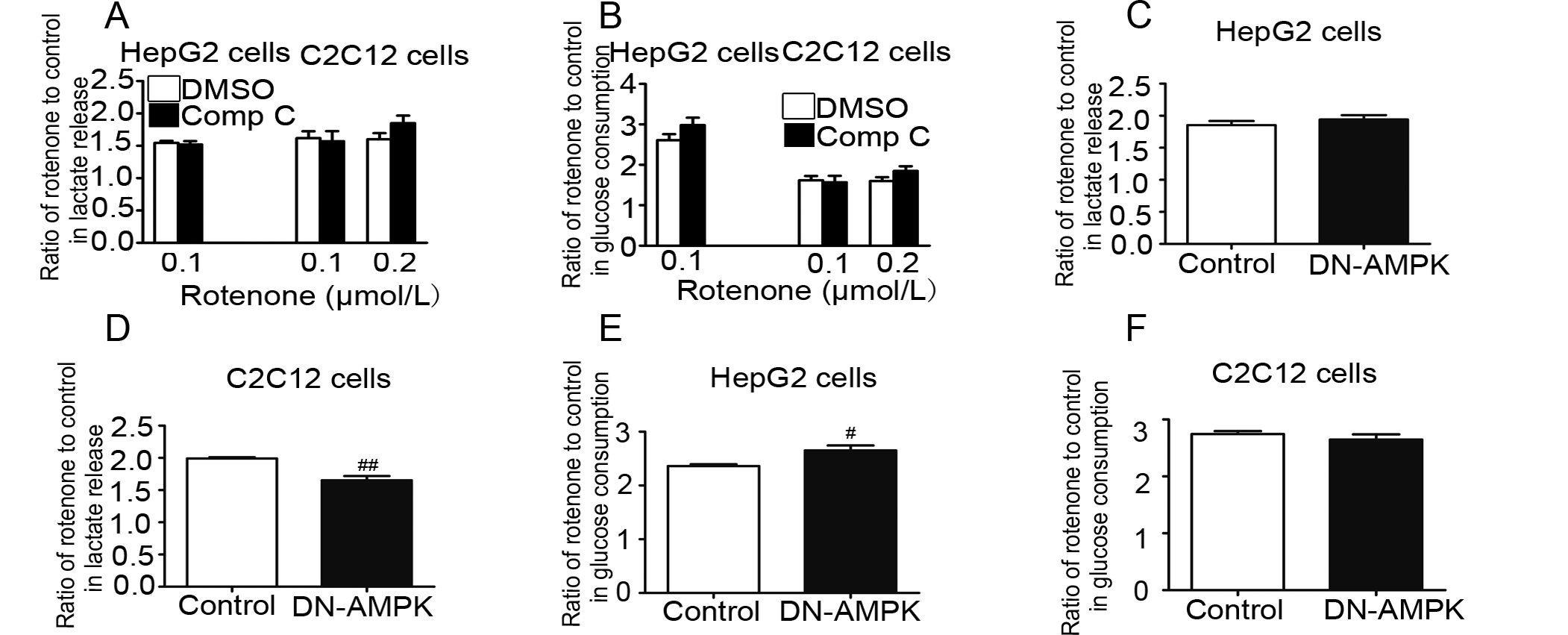
**

**Supplementary Figure 1.** The increasing rates of lactate release and glucose consumption stimulated by rotenone did not change with AMPK inactivation. The ratios of rotenone to control in lactate release (A) and glucose consumption (B) in HepG2 and C2C12cells treated with vehicle or compound C were compared. The ratios of rotenone to control in lactate release (C and D) and glucose consumption (E and F) were compared between the cells infected with adenoviruses expressing control GFP and those with DN-AMPK. Data are expressed as means ± SEM; n = 3-8 for each group. ^#^*P*< 0.05 and ^##^*P*< 0.01 *vs*. control.
